# BacSJ—Another Bacteriocin with Distinct Spectrum of Activity that Targets Man-PTS

**DOI:** 10.3390/ijms21217860

**Published:** 2020-10-23

**Authors:** Aleksandra Tymoszewska, Piotr Walczak, Tamara Aleksandrzak-Piekarczyk

**Affiliations:** Institute of Biochemistry and Biophysics, Polish Academy of Sciences (IBB PAS), Pawińskiego 5a, 02-106 Warsaw, Poland; tymoszewska@ibb.waw.pl (A.T.); pedrowalczak@gmail.com (P.W.)

**Keywords:** BacSJ, subclass IId bacteriocin, bacteriocin receptor, mannose-specific PTS (Man-PTS), *Lactococcus lactis*

## Abstract

Lactic acid bacteria produce diverse antimicrobial peptides called bacteriocins. Most bacteriocins target sensitive bacteria by binding to specific receptors. Although a plethora of bacteriocins have been identified, for only a few of them the receptors they recognize are known. Here, we identified permease IIC and surface protein IID, two membrane subunits of the mannose-specific quaternary phosphotransferase system (Man-PTS), as a receptor for BacSJ, a subclass IId bacteriocin produced by *Lactobacillus paracasei* subsp. *paracasei* BGSJ2-8. BacSJ shares 45% identity with another Man-PTS binding bacteriocin, garvicin Q (GarQ). Similarly to GarQ, BacSJ has a relatively broad activity spectrum acting against several Gram-positive bacteria, such as *Lactococcus lactis* and *Listeria monocytogenes,* harboring fairly similar Man-PTSs, but not against *Lactococcus garvieae*. To identify specific Man-PTS amino acids responsible for the *L.* *lactis* sensitivity to BacSJ, and thus likely involved in the interaction with this bacteriocin, we generated eight independent BacSJ resistant *L.* *lactis* mutants harboring five distinct missense mutations in the *ptnC* or *ptnD* genes encoding the IIC and IID subunits. Concurrently with the resistance to BacSJ, the mutants efficiently utilized mannose as a carbon source, which indicated functionality of their mutated Man-PTS. The amino acid substitutions in the mutants localized to the intracellular region of the IIC permease or to the extracellular parts of IID. This localization coincides with regions targeted by GarQ and some other Man-PTS-binding garvicins, pointing to similarities between all these bacteriocins in the mechanism of their interaction with Man-PTS. During the attack by these bacteriocins, subunits IID and IIC are assumed to function sequentially as a docking and an entry module allowing the toxic peptide to bind the cell and then open the pore. However, since not all of the BacSJ-resistant mutants exhibited cross-resistance to GarQ, we propose that BacSJ interacts with Man-PTS in a manner slightly different from that of GarQ.

## 1. Introduction

Ribosomally synthesized antimicrobial peptides or proteins, categorized as bacteriocins, provide the bacterial strains producing them with an advantage over susceptible bacterial strains competing for nutrients or habitats in the respective ecological niches [1]. Bacteriocins can kill or inhibit the growth of bacterial strains from closely related species (narrow-spectrum bacteriocins) or from distant genera (broad-spectrum bacteriocins). They have no activity against the producers’ cells which are protected by specific immunity proteins [1]. The bacteriocins of lactic acid bacteria (LAB) are especially interesting as they have been consumed by humans for centuries in fermented meat, vegetables, and dairy products, and are generally considered to be natural and safe for use [2]. Numerous studies have reported that bacteriocins or bacteriocin-producing strains may be used as biopreservatives to enhance the stability and safety of food [3]. In addition, bacteriocins have many properties suggesting that they can be applied as an alternative to clinically used antibiotics to fight infections caused by antibiotic-resistant pathogens [4]. Bacteriocin-producing probiotic strains may also be used with antibiotic therapy to promote a desirable intestinal microbiota and help eliminate pathogenic bacterial strains [5]. Some bacteriocins have also been shown to exhibit significantly higher apoptotic and necrotic activity against cancer cells, in comparison to normal cells, which makes them promising novel anticancer therapeutic agents [6].

Bacteriocins produced by LAB are diverse in terms of their genetic characteristics, structure, molecular weight, post-translational modifications, and physicochemical properties. Based on these criteria, several classifications with different numbers of classes or descriptions of subclasses have been proposed. In all of them, two main classes are distinguished: class I bacteriocins (lantibiotics) which are small, post-translationally modified peptides containing unusual amino acids such as lanthionine and 3-methyllanthionine, and class II bacteriocins which are larger, non-modified, or slightly modified peptides. Among the class II bacteriocins, usually four subclasses are recognized: subclass IIa—pediocin-like bacteriocins, subclass IIb—two-component bacteriocins, subclass IIc—circular bacteriocins, and subclass IId—linear, non-pediocin-like bacteriocins [7]. The majority of newly synthesized bacteriocins contain a leader sequence with a conserved double-glycine motif (GG-motif) which is cleaved off during export by a dedicated ABC transporter or the Sec system, giving a mature extracellular peptide [8,9]. However, a group of so-called leaderless bacteriocins, often classified as a separate subclass of class II bacteriocins [10], contain a formylated methionine instead of the leader sequence at the N-terminus [11].

Over the past years, an increasing number of class II bacteriocins have been shown to act through a receptor-dependent mechanism, i.e., to recognize and bind specific membrane targets (receptors) on the cell surface causing rapid disruption of the membrane eventually leading to cell death [12,13,14,15,16,17,18,19]. Among the few hitherto recognized bacteriocins receptors, mannose-specific phosphotransferase system (Man-PTS) seems to have an outstanding role. The main function of this four-component system is the uptake of mannose and glucose with their concurrent phosphorylation [20]. Beyond this principal function of Man-PTS, several studies demonstrated its interactions with subclass IIa bacteriocins (pediocins) [21,22,23]. Later, its receptor role was confirmed also for two subclass IId bacteriocins—lactococcin A (LcnA) and lactococcin B (LcnB) (LcnA-like bacteriocins; lactococcins) [24]. Most recently, a role of Man-PTS in the sensitivity to further subclass IId bacteriocins, such as lactococcin Z (LcnZ) [18] and garvicins ABC and Q (GarA, GarB, GarC, GarQ) has been shown [16,17]. All these bacteriocins exhibit a diversified spectrum of antibacterial activity, being highly potent against only single species or against wider range of bacteria. The first group includes LcnABZ (active only against *L. lactis* strains), GarABC (GarAB active against *L. garvieae* and GarC active against all *Lactococcus* spp.), whereas the latter one comprises pediocins and garvicin Q, both killing several bacterial species from numerous genera including, among others, *Carnobacterium, Enterococcus*, *Lactobacillus*, *Leuconostoc*, *Listeria,* and *Pediococcus*. In addition to their considerable dissimilarities in the antimicrobial activity spectrum, the Man-PTS-targeting bacteriocins also differ significantly in their amino acid sequence, suggesting distinct modes of interaction with Man-PTS. Among the four Man-PTS components, only the membrane-localized IIC and IID subunits have been proven to be indispensable for the binding of bacteriocins, whereas the two other components, intracellular IIA and IIB, are expendable [16,17,24]. Within the sequences of IICD, three distinct regions called α, β, and γ are recognized. Region α, crucial for the action of pediocins, lies in the N-terminal part of subunit IIC and is present in *L. monocytogenes* but absent in the genus *Lactococcus*. Region β, rich in glycine and essential for activity of all bacteriocins, is localized in the C-terminal part of subunit IIC, whereas region γ is in subunit IID [25]. The latter forms an extracellular loop of 35–40 amino acids except in *L. garvieae*, where an elongated region γ containing an additional part γ+ is present (17). In this species, γ+ is required for the antibacterial activity of GarABC [17]. Distinct amino acid residues located in the extracellular parts of the surface protein IID and in the transmembrane regions of the permease IIC are critical for the action of each of the GarABCQ bacteriocins [16,17].

Although the role of Man-PTS as a bacteriocin receptor is well documented, the exact mechanism of the bactericidal action of the Man-PTS-targeting bacteriocins is not fully understood. Numerous studies have shown that pediocins, lactococcin A, and lactococcin B form pores in the bacterial membranes. The pore formation results in an uncontrolled leakage of cytoplasmic metabolites such as ions and amino acids, depletion of intracellular ATP, dissipation of the proton-motive force, and consequently cell death [26,27,28,29]. Recent studies have shown high similarity between garvicins ABQ and transmembrane fragments of channel-forming proteins, implying that these peptides may also act by forming pores that facilitate the leakage of intracellular solutes [16,17]. However, it is still unclear whether the interaction of bacteriocins with Man-PTS induces structural changes in the permease that leads to the opening of the intrinsic channel in IIC, as has been proposed for garvicins, lactococcins and some pediocin-like bacteriocins [16,17,24], or whether bacteriocins only employ Man-PTS as a docking molecule to get closer to the membrane and then themselves form pores in the lipid bilayer as proposed for enterocin CRL35 [30,31]. Moreover, lactococcin Z, despite having a bactericidal activity, has been found to neither dissipate the membrane potential nor induce ATP efflux, indicating that its mechanism of action does not involve pore formation [18]. In addition, garvicin A was shown to act by inhibiting the cell wall biosynthesis, most probably septum formation [32]. Nevertheless, those authors emphasized that some bacteriocins may affect the formation of the septum when used at concentrations much higher than required for pore formation [32].

BacSJ is a subclass IId bacteriocin produced by *Lactobacillus paracasei* subsp. *paracasei* BGSJ2-8 isolated from semi-hard homemade cheese [33]. It is a peptide of 68 amino acids with an 18-amino acid leader with the conserved GG-motif. It is encoded by the *bacSJ2-8* gene on the pSJ2–8 plasmid together with the *bacSJ2-8i*, *abcT,* and *acc* genes encoding, respectively, the immunity protein, ABC transporter, and an accessory protein, all indispensable for the production of a functional bacteriocin [34]. It is heat-stable and active in a broad range of pH (from 2 to 11). BacSJ is active against several *L. paracasei* strains closely related to the producer strain, and an *L. lactis* strain [33].

In this study, we report the identification of subunits IIC and IID of Man-PTS as a receptor for BacSJ. We also demonstrate that BacSJ and GarQ show some amino acid sequence similarity, in contrast to other Man-PTS-binding bacteriocins such as garvicins ABC and lactococcins ABZ. Despite the partial similarity to GarQ, BacSJ has a unique activity spectrum and shows distinct mode of interaction with the receptor. Specific amino acids of the receptor likely involved in the interaction with BacSJ have been determined experimentally; they are localized in extracellular regions of IID and intracellular region of IIC. This report furthers the role of Man-PTS as a receptor for numerous non-homologous and homologous bacteriocins with a diverse spectrum of activity.

## 2. Results

### 2.1. BacSJ Homologs are Encoded in Some Lactobacillales

**Figure 1:**
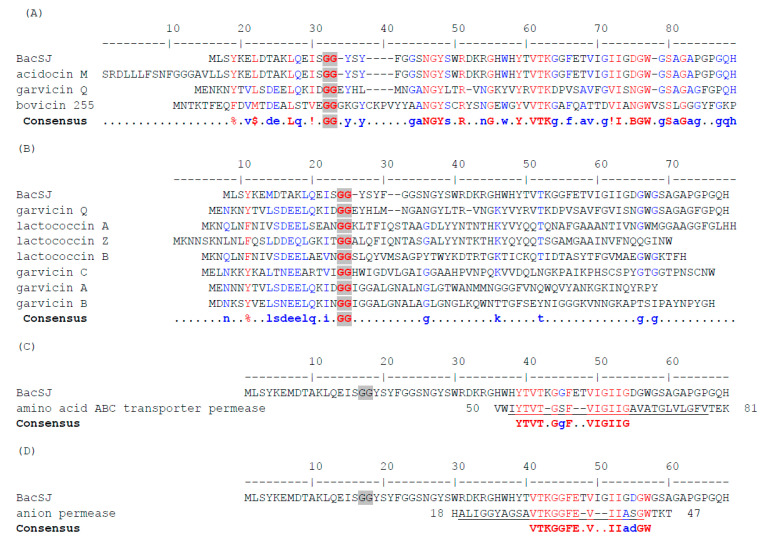
Alignment of the amino acid sequences of BacSJ prepeptide with its homologs (**A**), with bacteriocins that use mannose-specific phosphotransferase system (Man-PTS) as a receptor (**B**), with Branched Chain Amino Acid (BCAA) ATP-Binding Cassette (ABC) transporter permease (**C**), and with anion permease (**D**). Fully conserved residues are in red, partially conserved ones in blue. Consensus symbols are: !—I or V; $—L or M, %—F or Y, B—D or N. Double glycine (GG) motifs are highlighted by grey background. Transmembrane regions are underlined. NCBI RefSeq or GenBank Accession nos. and host organisms are: bacteriocin BacSJ—CAR92206.2, *Lactobacillus paracasei* subsp. *paracasei* BGSJ2-8, plasmid; acidocin M, partial—BAB86318.1, *Lactobacillus acidophilus* TK8912, plasmid; prepeptide GarQ—AEN79392.1, *Lactococcus garvieae* BCC 43578, plasmid; bovicin 255 peptide precursor—AAG29818.1, *Streptococcus* sp. LRC 0255, chromosome; lactococcin A—WP_015081786.1, lactococcin B—WP_015081788.1, *Lactococcus lactis* subsp. *cremoris* 9B4, plasmid; lactococcin Z precursor—BAU29928.1, *Lactococcus lactis* QU7, chromosome; garvicin A—WP_014386638.1, garvicin B—WP_014386584.1, garvicin C—WP_014386275.1, *Lactococcus garvieae* 21881, plasmids; BCAA ABC transporter permaese—QHE60941.1, *Bacillus vietnamensis*; anion permease, partial—OLD57848.1, *Acidobacteria bacterium*.

BacSJ is synthesized as a 68-amino acid prebacteriocin with the GG-motif and after processing gives a mature form of 50 amino acid (Figure 1). It shares similarity with 86 peptides (identity threshold 32%) encoded on plasmids or chromosomes of several species of the families *Enterococcaceae*, *Lactobacillaceae*, *Leuconostocaceae,* and *Streptococcaceae*, with the majority of representatives among *Lactobacillus* and *Streptococcus* spp. (Figure S1). Most of these peptides are annotated as hypothetical proteins or putative pheromones/bacteriocins; only two (garvicin Q and bovicin 255 produced by *Lactococcus garvieae* BCC 43,578 and *Streptococcus* sp. LRC 0255, respectively) have previously been assigned as functional bacteriocins [35,36], and one (acidocin M encoded on plasmid pLA103 of *Lactobacillus acidophilus* TK8912) as a protein of unknown function without an AUG start codon [37].

These homologous peptides usually contain a conserved processing site of double glycine (GG) and the cleaved off leader peptides are 10–33 amino acids long and show significant similarity within groups of similar mature peptides (Figure S1). The mature peptides share with BacSJ several amino acids in their N-terminal and central parts, with variable C-termini. The most notable motifs include the N-terminal NGY and central VTK (Figure S1), which are also present in the functionally characterized garvicin Q and bovicin 255 (Figure 1A) but absent in subclass IId bacteriocins that employ Man-PTS as a receptor (Figure 1B). The amino acid sequence of BacSJ shows the highest homology with that of acidocin M. 98.5% and 100% identities between prepeptides and predicted mature peptides of BacSJ and AcdM, respectively, are observed. BacSJ prepeptide also shares respectively 47% and 45% identity with prepeptides of Bov255 and GarQ, while BacSJ predicted mature form shares, respectively, 47% and 46% identity with predicted mature peptides of Bov244 and GarQ. A 16-amino acid fragment of BacSJ comprising the VTK motif is 75% identical to a transmembrane region of the Branched Chain Amino Acid (BCAA) ATP-Binding Cassette (ABC) transporter permease from *Bacillus vietnamensis* (Figure 1C). Almost the same fragment of 17 amino acids of BacSJ is 71% identical to a transmembrane region of an anion permease from *Acidobacteria bacterium* (Figure 1D).

**Figure 2:**
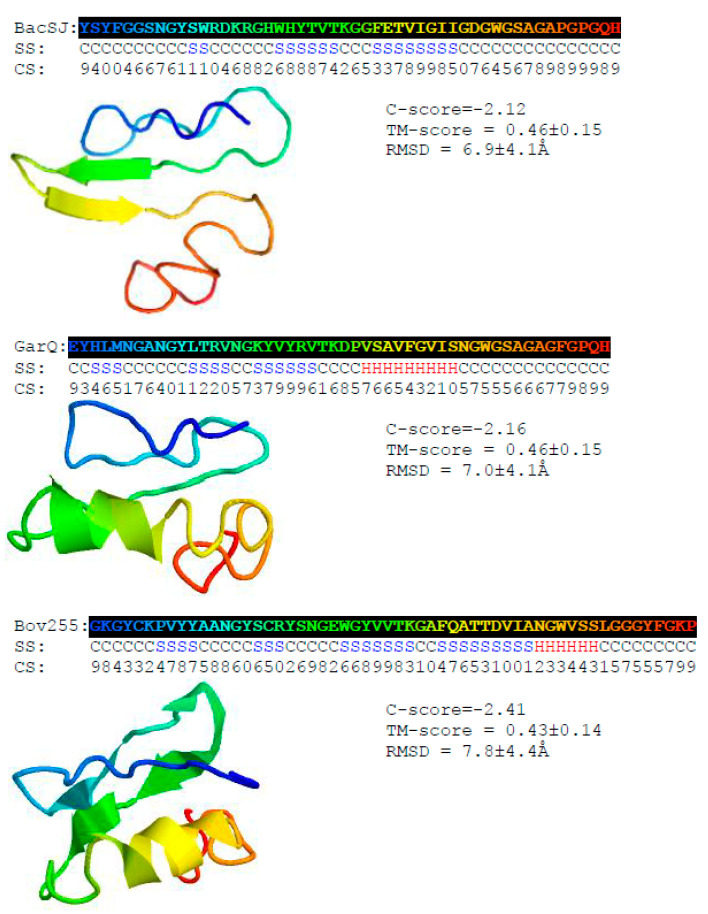
Predicted secondary and tertiary structures of BacSJ, Bov255, and GarQ. H, S, and C indicate helix, strand, and coil, respectively. The confidence score (CS) ranges from 0 to 9 and represents the certainty of the secondary structure (SS) prediction. C- and TM-scores estimate the global accuracy of the 3D structure model. C-score in the range from −5 to 2 and TM-score > −1.5 indicates a model with a correct global topology. Root mean square distance (RMSD) is the average distance of pairs of residues between model and template.

The predicted secondary structures of BacSJ, Bov255 and GarQ comprise several β-strands of two to nine amino acids in their central and N-terminal parts. Additionally, Bov255 and GarQ contain an α-helix of, respectively, six and nine amino acids at their N-termini. Among these three bacteriocins, BacSJ is the most disordered, with relatively long unstructured N- and C-termini. Template-based 3D models of the bacteriocins revealed globular structures relatively ordered in the central part and disordered in the N- and C-terminal regions (Figure 2).

### 2.2. BacSJ Has a Moderate Spectrum of Activity

The inhibitory activity of BacSJ was tested against a wide range of Gram-positive bacteria, some Gram-negative indicator strains, and yeast. Most of the Gram-positive bacteria tested, except for the genera *Bacillus*, *Streptococcus*, and *Staphylococcus*, were sensitive to BacSJ (Table 1). BacSJ also inhibited the growth of all *Lactobacillus* and *Lactococcus* species except for *Lactobacillus kunkeei* and *L. garvieae*. In the case of *Pediococcus* spp., the susceptibility to BacSJ was species-specific since it was active against *P. pentosaceus* but not *P. acidilactici* or *P. parvulus*. We also noted a significant antimicrobial action towards the pathogenic *Listeria monocytogenes*. In contrast, BacSJ showed no activity against any of the Gram-negative bacteria tested, nor the opportunistic pathogenic yeast *Candida albicans* (Table 1).

### 2.3. Man-PTS Subunits IIC and IID are Necessary for BacSJ Activity

**Figure 3:**
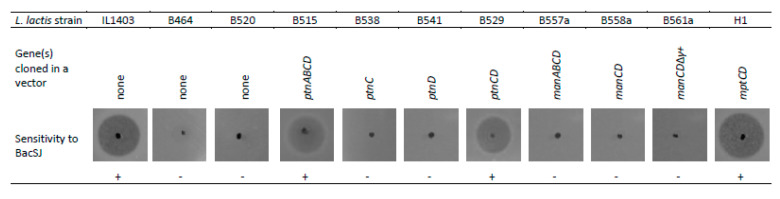
Restoration of BacSJ sensitivity of *L. lactis* Δ*ptnABCD* (*L. lactis* B464) by Man-PTS compounds from different species. The deleted *ptnABCD* operon in *L. lactis* B464 was complemented by expressing genes encoding indicated Man-PTS components from different species (*ptnABCD* from *L. lactis*, *manABCD* from *L. garvieae*, *mptCD* from *L. monocytogenes*). *L. lactis* IL1403 and B520 are control strains either wild-type or with *ptnABCD*-deletion carrying an empty vector. The genetic features of modified strains are described in Supplementary Table S1.

BacSJ shares 45% identity with GarQ (Figure 1A) which employs Man-PTS as a receptor in a broad range of bacterial species [16]. We therefore tested the sensitivity of an *L. lactis* B464 mutant with a deletion of the *ptnABCD* genes encoding Man-PTS to BacSJ and found it to be fully resistant, whereas the parental wild-type strain *L. lactis* IL1403 was sensitive (Figure 3). Complementation of that deletion in *L. lactis* B515 (with a complete *ptnABCD* operon) or *L. lactis* B529 (with *ptnCD* genes only) restored the BacSJ sensitivity fully (Figure 3). The same effect was observed when the *mptCD* genes encoding membrane components of listerial Man-PTS were expressed in *L. lactis* B464 (strain H1). On the other hand, expression of *ptnC* or *ptnD* genes alone was not sufficient to cause sensitivity to BacSJ (strains B538 and B541, respectively) (Figure 3).

BacSJ is active against all *L. lactis* strains tested but shows no activity towards their close relative *L. garvieae* (Table 1) in which Man-PTS is encoded by the *manABCD* genes. In accordance, neither *manCD* (strain B558a) nor *manABCD* (strain B577a) reverted the resistance of *L. lactis* B464 to BacSJ (Figure 3). Interestingly, the Man-PTS systems of the two species are highly similar and their subunits IIC and IID share, respectively, 79% and 62% of identity. The most notable difference between these two systems is the presence of an additional 51-amino acid loop (so-called region *γ*+) in the extracellular part of *L. garvieae* subunit IID. Therefore, we reasoned that this additional sequence somehow prevents the sensitivity of *L. garvieae* to BacSJ. To verify this assumption, we tested an *L. lactis* B561a mutant with the nucleotides encoding the *γ*+ region removed. After the removal of γ+ loop, the identity of IID between *L. garvieae* and *L. lactis* increased to 72%, but unexpectedly, the strain was still resistant to BacSJ (Figure 3).

### 2.4. Targeted Mutagenesis Allows Identification of Man-PTS Amino Acids Needed for BacSJ Activity

To identify the amino acids of Man-PTS recognized by BacSJ we applied the method used successfully earlier involving generation of bacteriocin-resistant mutants with amino acid substitutions in the IIC or IID and maintained functionality of the Man-PTS sugar transport system [16,17]. Presence of BacSJ in growth medium with mannose allowed obtaining eight *L. lactis* mutants with an at least 8-fold decreased sensitivity to BacSJ (M3, M6, M8, M9, M16, M19, M30, M33) (Table 2). Sequencing of their *ptnABCD* operon showed the presence of five independent missense mutations leading to the substitutions of Gly62→Val in IIC or Arg200→His, Leu83→Phe, Phe226→Ser, or Leu197→Phe in IID (Table 2). We also tested the sensitivity to BacSJ of the GarQ-resistant *L. lactis* LLN1 mutant with the Pro123 →His substitution in IID obtained earlier [16]. In addition, that substitution decreased the sensitivity to BacSJ (Table 2). On the other hand, not all BacSJ-resistant mutants exhibited cross-resistance to garvicin Q. One of them, carrying the Leu83→Phe substitution in subunit IID, was still fully susceptible to GarQ. The other BacSJ-resistant mutants also showed an 8–64-fold decreased sensitivity to garvicin Q in comparison to the wild-type strain (Table 2).

**Figure 4:**
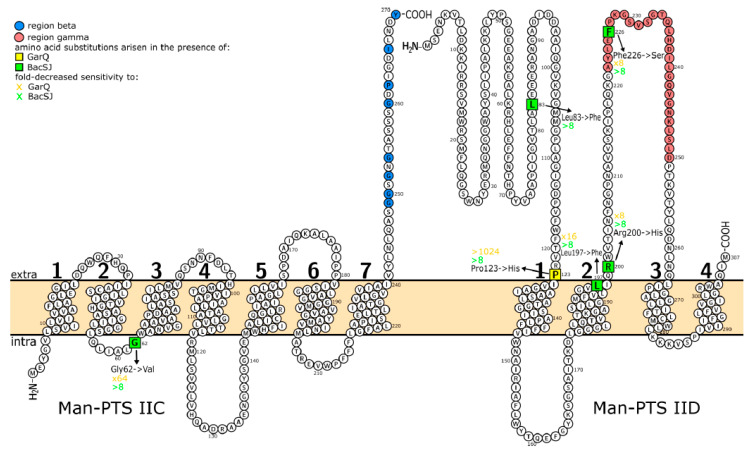
Predicted membrane topology of *L. lactis* IL1403 Man-PTS subunits IIC and IID. Amino acids substituted in spontaneous bacteriocin-resistant mutants are marked with squares. Numbers indicate successive transmembrane regions.

The obtained substitutions localized to distinct parts of Man-PTS in relation to the bacterial membrane, i.e., to its outer, inner, or transmembrane regions. Thus, the only mutation in the IIC subunit (Gly62→Val in mutants M3, M8, M9) occurred in the intracellular loop next to the third transmembrane region (Figure 4). Most of the amino acid substitutions in IID appeared in the outside regions—one in the N-terminus and two in the extracellular loop harboring the γ region. Finally, the Leu197→Phe substitution is located near the cell surface, in the second transmembrane region (Figure 4). To determine whether the substituted amino acids are conserved among other BacSJ-sensitive species, we compared the Man-PTS subunits IICD of the 24 strains that were used to determine the bacteriocin activity spectrum. For 16 of those strains, the relevant sequences were determined in our previous study [16] and the remaining eight are available in the GenBank (accession numbers are included in Supplementary Table S1). Four of the five amino acids substituted in the BacSJ-resistant mutants (Gly62 in IIC, Leu83, Leu197, and Arg200 in IID) were fully conserved in all BacSJ-sensitive species as well as in the BacSJ-resistant *L. garvieae.* The remaining Phe226 was conserved only among *L. lactis* while in other species, including BacSJ-resistant *L. garvieae*, a Trp residue was present at this site (Table 3). In addition, the intracellular localization of Gly62 and extracellular of Leu83, Arg200, and Phe226 in the predicted Man-PTS topology were highly conserved in all 24 strains. Only Leu197 was equally often localized in the transmembrane and outer Man-PTS regions (Table 3).

## 3. Discussion

The mannose-specific phosphotransferase system Man-PTS consists of four components of which two (IIC and IID) are embedded in the bacterial membrane. Its main function is the uptake and concomitant phosphorylation of a broad range of sugars, which apart from mannose, also include glucose, fructose, and glucosamine. Man-PTS has also been found to mediate the lethal effect of some bacteriocins [16,17,18,24]. Here, we showed that also BacSJ, a subclass IId bacteriocins acts by binding to the IICD components of Man-PTS.

To identify a potential BacSJ receptor, we first performed extensive homology searches to find that BacSJ homologs are widespread among species of the genera *Enterococcaceae*, *Lactobacillaceae*, *Leuconostocaceae,* and *Streptococcaceae*. The highest homology was found with a protein of unknown function from *L. acidophilus* TK8912 annotated as acidocin M. A high similarity between the pSJ2–8 plasmid from *L. paracasei* subsp. *paracasei* BGSJ2–8 encoding BacSJ and encoding AcdM pLA103 plasmid has been noted earlier suggesting possible horizontal gene transfer [34]. A comparison of the genes encoding these two bacteriocins shows an A→ T substitution in the initial ATG codon of the *acdM* gene causing the lack of the N-terminal methionine and instead, the creation of UUG-Leu. Despite the fact that some Prokaryotes may use UUG-Leu as an alternate start codon [38], it seems that this is not the case in *L. acidophilus* TK8912, since according to Tahara et al. (1992), acidocin M was not purified from the growth medium [39]. It suggests that despite the 100% identity between the predicted amino acid sequences of mature BacSJ and AcdM, the latter is not produced due to the lack of its translation. Other homology searches revealed that BacSJ shares some identity with Bov255 and GarQ. Of those two, only GarQ has been proven to use Man-PTS as a receptor [16]. To verify whether Man-PTS is also targeted by BacSJ, we tested the bacteriocin sensitivity of an *L. lactis* mutant lacking the Man-PTS-encoding genes. The full resistance of the deletion mutant confirmed that Man-PTS is essential for BacSJ action. Moreover, complementation with lactococcal or listerial genes encoding only the IICD components of Man-PTS showed that these membrane subunits are sufficient to confer sensitivity to BacSJ. The overall homology of Bov255 with BacSJ and GarQ and the presence of conserved motifs in all these bacteriocins suggest that Bov255 may be yet another bacteriocin employing the IICD components of Man-PTS as a receptor. To confirm this assumption unequivocally, the effects of a deletion and complementation of Man-PTS-encoding genes in *L. lactis* on its sensitivity to Bov255 must be determined.

Originally, BacSJ was reported to be active only against several *L. paracasei* strains and a single *L. lactis* strain, suggesting its rather narrow activity spectrum [33]. Here, we significantly expanded the range of strains tested and found that, in fact, BacSJ has a relatively broad spectrum of activity, being bactericidal against many species from several Gram-positive bacterial genera such as *Enterococcus*, *Lactobacillus*, *Lactococcus*, *Listeria*, and *Pediococcus*, all harboring a functional Man-PTS. Importantly, BacSJ is also active against *Listeria monocytogenes*, a food-borne pathogen causing listeriosis in both human and animals [40]. The earlier-known bacteriocins that bind to Man-PTS have highly diversified activity spectra and include narrow-spectrum subclass IId bacteriocins such as GarABC [17] and LcnABZ [18,24] as well as the wide-spectrum GarQ [16] and subclass IIa pediocins [24]. This study shows that the activity spectrum of BacSJ is nearly identical to that of GarQ [16]. Among 70 strains tested, the only difference in their sensitivity to BacSJ and GarQ activity was found for *L. garvieae* which was resistant to BacSJ and highly susceptible to GarQ. Interestingly, based on the reported results it can be assumed that also Bov255 has a relatively broad spectrum of activity limited to Gram-positive bacteria [36]. It would be relevant to determine its activity against a larger panel of strains including *L. garvieae* and then to classify it within the GarQ-like or BacSJ-like group of bacteriocins. *L. garvieae* is a close relative to *L. lactis*, nevertheless, *L. garvieae* is resistant to BacSJ and sensitive to GarQ, whereas *L. lactis* is sensitive to both these bacteriocins. Since the component IID of *L. garvieae* harbors an additional 51 amino acid long extracellular loop (so-called region γ+), which is absent in other species including *L. lactis* [17], we assumed that this structure could have a decisive role in the resistance to BacSJ. To prove it, we removed region γ+ from *L. garvieae* thereby recreating a typical γ loop. Unexpectedly, after that removal the deletion mutant did not become sensitive to BacSJ, suggesting that the resistance of *L. garvieae* to BacSJ is due to reasons other than the presence of the γ+ loop.

The differences between BacSJ and the other bacteriocins that use Man-PTS as a receptor also concern their structures—although BacSJ shares 45% identity with GarQ, there is virtually no similarity to the remaining bacteriocins from this group. This refers to both their primary as well as the predicted tertiary structures, suggesting that BacSJ may use a novel bacteriocin—receptor binding mode. Here, we specified individual amino acids of Man-PTS likely involved in the interaction with BacSJ since their substitution led to a resistance of the respective *L. lactis* mutants. A predicted IICD membrane topology indicated that these amino acids are localized in outer regions of IID easily accessible for the bacteriocin (Leu83, Pro123, Arg200, Phe226) or adjacent to the outer membrane (Leu197). In contrast, the only amino acid of IIC possibly involved in the interaction with BacSJ found now, Gly62, is located in an intracellular loop, next to the inner membrane. Most of these amino acids also seem to have a role in the binding of GarQ, consistent with a general cross-resistance between these two bacteriocins, but a single exception can be found—the Leu83→Phe substitution leading to BacSJ resistance has no effect on the *L. lactis* sensitivity to GarQ. Moreover, the Met34→Ile and Ala58→Asp substitutions in IID of *L. garvieae* obtained in our previous study [17] had minimal or no effect on the sensitivity to GarQ. Since all these three amino acids are located near the N-terminus of IID, this part of the extracellular region of IID seems dispensable for GarQ and essential for BacSJ binding, indicating that specific regions of IID are involved in the binding of individual bacteriocins. Altogether, the obtained results confirm that BacSJ uses a novel binding mode, distinct from that used by GarQ and other Man-PTS-targeting bacteriocins, to dock to the *L. lactis* cell. Nevertheless, it cannot be excluded that the amino acids in question are in fact not directly engaged in the interaction with bacteriocin but instead play an important role in determining the Man-PTS structure and therefore their mutation affects the Man-PTS structure such that it does not compromise the sugar transport function but prevents bacteriocin binding.

A comparison of the amino acid sequences of IICD from different bacterial species showed that the residues mutated in the BacSJ-resistant *L. lactis* mutants are highly conserved among BacSJ-sensitive species. The only exception was Phe226 found exclusively in *L. lactis* and replaced by tryptophan in other bacteria. Since both these residues are aromatic and hydrophobic, they could behave similarly in the interaction with BacSJ. Notably, the conserved amino acids were also present in the BacSJ-resistant *L. garvieae* but we propose that they were inaccessible for BacSJ as a consequence of the altered IICD structure due to the presence of the additional γ+ region. Specifically, *L. garvieae* Leu200, Arg203, and Trp230, equivalents of *L. lactis* Leu197, Arg200, and Phe226, lie, respectively, 33, 30, and 3 amino acids from region γ+. One should note, however, that deletion of γ+ failed to make *L. garvieae* sensitive to BacSJ, suggesting that those amino acids alone may not be sufficient for the bacteriocin activity. In addition, the localization of the supposedly critical amino acids and their counterparts in predicted Man-PTS membrane topology seems highly conserved among distinct species. In our previous study, we predicted the tertiary structure of IICD from *L. garvieae* [17]. Although BacSJ has no activity against this species, all four equivalents of substituted amino acids in *L. garvieae* IID localize in outside regions and the homolog of Gly62 in IIC is situated in a intracellular region which corresponds to the localization of substituted amino acids in the predicted membrane topology of IICD from *L. lactis*. To determine their spatial localization in the *L. lactis* Man-PTS and gain a deeper insight into the possible Man-PTS-BacSJ interactions we undertook to predict the tertiary structure of *L. lactis* IICD using homology modeling. Due to a lack of an appropriate threading templates we failed to structure IID reliably; however, using the structure of cation-bound Multidrug and Toxin Compound Extrusion (MATE) transporter (PDB ID 3MKT) as a template of the highest significance we did model IIC. In this 3D model (not shown), the distribution of intracellular, transmembrane, and extracellular regions corresponded well with the predicted two-dimensional topology of IIC. In addition, the location of the altered Gly62 next to the inner cell membrane was confirmed.

The overall location of the amino acids predicted to interact with BacSJ is similar to that of the ones targeted by Man-PTS-binding garvicins. Gly52 and Leu59 in IIC from *L. garvieae* that are essential for the sensitivity to GarABCQ [17] are localized, respectively, in the second transmembrane region and in the following intracellular loop, as is Gly62 in IIC of *L. lactis*. Likewise, most of the IID residues important for the sensitivity of *L. garvieae* to GarBCQ [17] are part of the same N-terminal region or extracellular loop that harbor Leu83, Arg200 and Phe226 in *L. lactis*. In contrast, no amino acids responsible for the sensitivity to BacSJ or the Man-PTS-specific-garvicins have been mapped to the transmembrane, intracellular, or C-terminal regions of IID, or the N- and C-terminal tails, extracellular, or most intracellular regions of IIC, indicating that all these regions likely have no direct role in the interaction with these bacteriocins. Such similarities in the distribution of residues potentially involved in binding BacSJ and garvicins suggest a similar mechanism of the attack of these bacteriocins on Man-PTS. We proposed such a mechanism earlier [17]. It assumes that the surface protein IID serves as a docking module in which the extracellular N-terminal and gamma regions provide the initial contact with bacteriocin. The bacteriocin binding induces conformational changes in IID resulting in opening of the channel of the IIC permease. Subsequently, the bacteriocin binds intracellular fragments of the opened IIC channel and stabilizes its open conformation, thereby causing an uncontrolled leakage of ions, amino acids and other cytoplasmic metabolites, and eventually cell death. This concept is further supported by the fact that the C-terminal fragment of BacSJ is significantly similar to transmembrane regions of transporters common across Gram-negative and Gram-positive bacteria—Branched-Chain Amino Acid (BCAA) ABC transporter permease and anion permease. The BCAA ABC transporters allow the uptake of amino acids such as leucine, isoleucine, and valine playing important roles in bacterial physiology, from protein biosynthesis to signaling and adaptation to amino acid starvation [41]. Anion permeases classified in the inorganic phosphate transporter family carry out the uptake of inorganic phosphate or inorganic sulfate by proton or sodium symport [42]. It seems feasible that while the N-terminal part of BacSJ interacts with extracellular region(s) of the IID protein, the C-terminal fragment of the bacteriocin forms a stable pore in the permease IIC.

In summary, this study reports the identification of BacSJ as a subclass IId bacteriocin that uses membrane subunits IIC and IID of Man-PTS as a receptor. To the best of our knowledge, BacSJ is the first broad spectrum bacteriocin recognizing Man-PTSs from *L. lactis*, but not from *L. garvieae* strains. BacSJ interacts with the receptor through a novel binding pattern, different from that used by other Man-PTS-targeting bacteriocins, which involves recognition of specific amino acids in IICD. Despite employing a unique amino acid binding pattern, we postulate that the mode of action of BacSJ is similar to that of Man-PTS-targeting garvicins and involves binding to the outer regions of IID and formation of an open pore with transmembrane or inner regions of IIC. Moreover, this study shows that homology analyses can effectively be used to assign bacteriocins to their receptors thereby reducing the number of bacteriocins with unknown receptors. In this context Man-PTS seems unique, being a receptor for multiple, homologous and non-homologous, bacteriocins. Expanding the family of Man-PTS-interacting bacteriocins and identification of differences in their mode of interaction with this receptor will be the focus of our studies.

## 4. Materials and Methods

### 4.1. Bacterial and Yeast Strains and Growth Conditions

The bacterial and yeast strains used in this study are described in Supplementary Table S1. Indicator strains were grown in Brain Heart Infusion (BHI) medium (Oxoid, Hampshire, UK) except *Campylobacter* strains that were grown in Blood Agar Base No. 2 (Oxoid). *L. lactis* IL1403-derived strains with deletion or complementation of the *ptnABCD* operon were grown in BHI medium, while *L. lactis* IL1403-derived strains with missense mutations in the *ptnABCD* operon were grown in Chemically Defined Medium (CDM) [43] supplemented with 1% mannose (man-CDM). Bacterial strains from the genera *Bacillus*, *Enterococcus*, *Lactococcus*, *Leuconostoc*, *Pediococcus, Streptococcus*, and the yeast *Candida albicans* were cultured at 30 °C under aerobic conditions without shaking. *Lactobacillus* were cultured at 37 °C under anaerobic conditions without shaking. Bacterial strains from the genera *Escherichia* and *Staphylococcus*, *Listeria monocytogenes*, *Pseudomonas aeruginosa*, and *Salmonella typhimurium* were cultured at 37 °C under aerobic conditions with shaking. *Campylobacter* were cultured at 37 °C under microaerobic conditions without shaking. *Carnobacterium maltaromaticum* was cultured at 16 °C under aerobic conditions without shaking. When appropriate, erythromycin and/or chloramphenicol (each at a concentration of 5 µg/mL) were added to the growth medium. Transcription of the genes encoding lactococcal (*ptnABCD* or *manABCD*) and listerial (*mptCD*) Man-PTSs, cloned in pNZ8037 under the nisin-responsive promoter, was induced by the addition of nisin to a concentration ranging from 10 to 50 ng/mL. Agar (Merck, Darmstadt, Germany) was added to 0.75% or 1.5% to the liquid medium to prepare soft agar (soft BHI-agar, soft man-CDM-agar) or agar plates (BHI-agar, man-CDM-agar), respectively.

### 4.2. Bacteriocin Preparation

Lyophilized BacSJ and GarQ with a purity of over 90% were chemically synthesized (PepMic, Suzhou, P.R. China) and dissolved before use to 1 mg/mL in 0.1% trifluoroacetic acid (TFA) (Sigma, Darmstadt, Germany).

### 4.3. Activity Spectrum Assay and Selection of Resistant Mutants

The inhibitory spectrum of BacSJ was determined and resistant *L. lactis* IL1403 missense mutants in the *ptnABCD* operon were obtained as described before [16]. To determine the inhibitory spectrum, 5 µL of BacSJ (1 mg/mL) was applied on BHI-agar plates. To generate spontaneous BacSJ resistant mutants, 5 mL of soft man-CDM-agar containing 100 µL of *L. lactis* IL1403 o/n culture and BacSJ at concentration of 0.06 mg/mL was poured onto a man-CDM-agar plates. Subsequently, the plates were incubated at 30 °C until single colonies appeared.

### 4.4. DNA Isolation and Manipulation

Genomic DNA was isolated using Genomic Mini Kit (A&A Biotechnology, Gdynia, Poland). Samples for *ptnCD* sequencing were prepared by PCR with *ptnC*for/rev and *ptnD*for/rev primers (Supplementary Table S1) using as a template the genomic DNA of *L. lactis* IL1403 and its mutants. PCR reactions were carried out in a final volume of 50 µL with the Phusion polymerase and Phusion HF Buffer (Fisher-Thermo Scientific, Waltham, MA, USA). The PCR cycling parameters were 98 °C for 30 s, (98 °C for 10 s, 55 °C for 30 s, 72 °C for 30 s) × 30 cycles, 72 °C for 5 min, and 4 °C for infinite. PCR products were purified using Wizard^®^ SV Gel and PCR Clean-Up System (Promega, Madison, WI, USA) and sequenced using Sanger method [44]. The data were analyzed using Clone Manager software (Sci-Ed, Westminster, CO, USA). Nucleotide sequences of the wild-type and mutated *ptnCD* genes were translated to amino acid sequences using the Translate tool on the ExPasy online server [45] (https://www.expasy.org/translate/). Protein sequences were aligned using the MultAlin online software [46] (http://multalin.toulouse.inra.fr/multalin/). Transmembrane regions of Man-PTS subunits IIC and IID, branched-chain amino acid ABC transporter permease, and anion permease were predicted with the HMMTOP automatic server [47] (http://www.enzim.hu/hmmtop/). Predicted topology of Man-PTS IICD was visualized using the Protter tool [48] (http://wlab.ethz.ch/protter/). BacSJ sequence homology searches were performed using the BLAST algorithm on the NCBI platform (https://blast.ncbi.nlm.nih.gov/Blast.cgi/). Tertiary structures of BacSJ, bovicin 255 (Bov255), and GarQ were predicted with the I-TASSER web service [49] (https://zhanglab.ccmb.med.umich.edu/I-TASSER/) using structural templates from the Protein Data Bank archive (PDB). Tailspike protein 3 (TSP3) from bacteriophage CBA120 (PDB ID 5W6F), prebacteriocin carnobacteriocin B2 (PDB ID 1RY3) and bacteriocin sakacin P in lipid micelles were used as templates of the highest significance for BacSJ, Bov255 and GarQ, respectively. The three-dimensional BacSJ structure was visualized using the PyMOL Molecular Graphics System, Version 2.0 (Schrödinger, LLC; https://pymol.org/2/) while Bov255 and GarQ structures were visualized using the open-source java-based molecule viewer Jmol (http://jmol.sourceforge.net/).

## Figures and Tables

**Table 1:** Inhibitory spectrum of BacSJ.

Group	Indicator Strain	BacSJ Activity
Fungi	*Candida albicans* CAI-4	−
G−	*Campylobacter jejuni* 12	−
G−	*Campylobacter jejuni* 480	−
G−	*Campylobacter jejuni* 81176	−
G−	*Campylobacter coli* 23/1	−
G−	*Escherichia coli* EC1000	−
G−	*Escherichia coli* TG1	−
G−	*Pseudomonas aeruginosa* ATCC 9027	−
G−	*Salmonella typhimurium* TT622	−
G+	*Bacillus cereus* IBB3390	−
G+	*Bacillus subtilis* BSB1	−
G+	*Carnobacterium maltaromaticum* IBB3447	+
G+	*Enterococcus durans* IBB3441	+
G+	*Enterococcus faecalis* IBB3439	+
G+	*Enterococcus faecalis* IBB3444	+
G+	*Enterococcus faecium* LMGT 2783	+
G+	*Enterococcus faecium* LMGT 2787	+
G+	*Lactobacillus johnsonii* IBB3155	+/−
G+	*Lactobacillus kunkeei* AH1	−
G+	*Lactobacillus kunkeei* AH38	−
G+	*Lactobacillus kunkeei* AH119	−
G+	*Lactobacillus paracasei* IBB3418	+
G+	*Lactobacillus paracasei* IBB3425	+
G+	*Lactobacillus paracasei* IBB3426	+
G+	*Lactobacillus paracasei* IBB3427	+
G+	*Lactobacillus paracasei* IBB3428	+
G+	*Lactobacillus paracasei* LOCK 0919	+
G+	*Lactobacillus paracasei* subsp. *paracasei* IBB3423	+
G+	*Lactobacillus paraplantarum* IBB3438	+
G+	*Lactobacillus plantarum* NC8	+
G+	*Lactobacillus plantarum* WCSF1	+
G+	*Lactobacillus plantarum* IBB3433	+
G+	*Lactobacillus plantarum* IBB3436	+
G+	*Lactobacillus plantarum* subsp. *plantarum* IBB3434	+
G+	*Lactobacillus rhamnosus* GG	+
G+	*Lactobacillus rhamnosus* IBB3429	+
G+	*Lactobacillus rhamnosus* LOCK 0900	++
G+	*Lactobacillus rhamnosus* LOCK 0908	+
G+	*Lactobacillus salivarius* IBB3154	+
G+	*Lactococcus garvieae* IBB3403	−
G+	*Lactococcus garvieae* IBB66	−
G+	*Lactococcus lactis* IBB3404	+
G+	*Lactococcus lactis* IBB3411	+
G+	*Lactococcus lactis* QU5 LMGT 3419	+
G+	*Lactococcus lactis* subsp. *cremoris* IBB3409	+
G+	*Lactococcus lactis* subsp. *lactis* IBB3407	+
G+	*Lactococcus lactis* subsp. *lactis* IL1403	+
G+	*Lactococcus raffinolactis* IBB91	+
G+	*Lactococcus lactis* IBB3446	+
G+	*Leuconostoc mesenteroides* IBB3442	+
G+	*Leuconostoc mesenteroides* IBB3443	+
G+	*Listeria monocytogenes* EDG-e	+
G+	*Pediococcus acidilactici* LMGT 2002	−
G+	*Pediococcus parvulus* IBB3448	−
G+	*Pediococcus pentosaceus* IBB3369	+
G+	*Staphylococcus aureus* ATCC 6538	−
G+	*Staphylococcus caprae* DSM-20608	−
G+	*Staphylococcus delphini* DSM-20771	−
G+	*Staphylococcus epidermidis* DSM-20044	−
G+	*Staphylococcus hyicus* DSM-20459	−
G+	*Staphylococcus intermedius* DSM-20373	−
G+	*Staphylococcus lugdunensis* DSM-4804	−
G+	*Staphylococcus pseudintermedius* DSM-21284	−
G+	*Staphylococcus saprophyticus* DSM-18669	−
G+	*Staphylococcus schleiferi* DSM-6628	−
G+	*Streptococcus agalactiae* IBB123	−
G+	*Streptococcuss agalactiae* IBB130	−
G+	*Streptococcus mitis* IBB3449	−
G+	*Streptococcus parauberis* IBB272	−
G+	*Streptococcus sobrinus* IBB3450	−

G− and G+ indicate Gram-negative and Gram-positive bacteria, respectively; “−”, no inhibition zone (strain resistance); “+/−”, minimal, vague inhibition zone (moderate strain sensitivity); “+” and “++”, wide, clear inhibition zone with, respectively, diameter ≤ 10 mm and ≥10 mm (strain sensitivity).

**Table 2:** Amino acid substitutions in Man-PTS from *L. lactis* IL1403 mutants resistant to BacSJ.

Mutant	Mutation	Amino Acid Change	Sensitivity to (Fold-Decreased Relative to WT)		Position in the Cell Membrane of Substituted Man-PTS Amino Acid
			GarQ	BacSJ	
GarQ-resistant mutant					
LLN1	C368→ A in *ptnD*	Pro123 → His	>1024×	>8×	outside
BacSJ-resistant mutants					
M3, M8, M9	G185 → T in *ptnC*	Gly62→Val	64×	>8×	inside
M6, M33	G599→ A in *ptnD*	Arg200 → His	8×	>8×	outside
M16	C247→T in *ptnD*	Leu83→Phe	0×	>8×	outside
M19	T677→C in *ptnD*	Phe226→Ser	8×	>8×	outside
M30	G591→ T in *ptnD*	Leu197→Phe	16×	>8×	transmembrane

**Table 3:** Alignment of Man-PTS IICD amino acids substituted in *L. lactis* IL1403 mutants resistant to BacSJ with their counterparts in different species.

Strain		IIC	IID			
BacSJ sensitive	*L. lactis* IL1403	Gly62 _i_	Leu83 _o_	Leu197 _t_	Arg200 _o_	Phe226 _o_
	*L. lactis* IBB3407	Gly62 _i_	Leu83 _o_	Leu197 _t_	Arg200 _o_	Phe226 _o_
	*L. lactis* IBB3409	Gly62 _i_	Leu83 _o_	Leu197 _t_	Arg200 _o_	Phe226 _o_
	*L. lactis* IBB2955	Gly62 _i_	Leu83 _o_	Leu197 _t_	Arg200 _o_	Phe226 _o_
	*L. lactis* LMGT3419	Gly62 _i_	Leu83 _o_	Leu197 _t_	Arg200 _o_	Phe226 _o_
	*L. paracasei* IBB3418	Gly62 _i_	Leu82 _o_	Leu196 _o_	Arg199 _o_	Trp222 _o_
	*L. paracasei* IBB3424	Gly62 _i_	Leu82^o^	Leu196^o^	Arg199^o^	Trp222^o^
	*L. paracasei* IBB3427	Gly62 _i_	Leu82 _o_	Leu196 _o_	Arg199 _o_	Trp222 _o_
	*L. paracasei* LOCK 0919	Gly62 _i_	Leu82 _o_	Leu196 _o_	Arg199 _o_	Trp222 _o_
	*L. paraplantarum* IBB3438	Gly62 _i_	Leu83 _o_	Leu197 _t_	Arg200 _o_	Trp224 _o_
	*L. plantarum* IBB3036	Gly62 _i_	Leu88 _o_	Leu202 _o_	Arg205 _o_	Trp229 _o_
	*L. plantarum* IBB3434	Gly62 _i_	Leu88 _o_	Leu202 _t_	Arg205 _o_	Trp229 _o_
	*L. plantarum* IBB3436	Gly62 _i_	Leu83 _o_	Leu197 _t_	Arg200 _o_	Trp224 _o_
	*L. plantarum* NC8	Gly62 _i_	Leu88 _o_	Leu202 _o_	Arg205 _o_	Trp229 _o_
	*L. plantarum* WCFS1	Gly62 _i_	Leu83 _o_	Leu197 _t_	Arg200 _o_	Trp224 _o_
	*L. rhamnosus* GG	Gly62 _i_	Leu82 _o_	Leu196 _o_	Arg199 _o_	Trp222 _o_
	*L. rhamnosus* IBB3429	Gly62 _i_	Leu82 _o_	Leu196 _o_	Arg199 _o_	Trp222 _o_
	*L. rhamnosus* LOCK 0900	Gly62 _i_	Leu82 _o_	Leu196 _o_	Arg199 _o_	Trp222 _o_
	*L. rhamnosus* LOCK 0908	Gly62 _i_	Leu82 _o_	Leu196 _o_	Arg199 _o_	Trp222 _o_
	*L. salivarius* IBB3154	Gly60 _i_	Leu83 _o_	Leu197 _o_	Arg200 _o_	Trp223 _o_
	*E. faecium* LMGT2783	Gly60 _i_	Leu83 _o_	Leu197 _o_	Arg200 _o_	Trp223 _o_
	*L. monocytogenes* EGD-e	Gly60 _i_	Leu82 _o_	Leu196 _t_	Arg199 _o_	Trp222 _o_
BacSJ resistant	*L. garvieae* IBB66	Gly60 _i_	Leu86 _o_	Leu200 _t_	Arg203 _o_	Trp230 _o_
	*L. garvieae* IBB3403	Gly60 _i_	Leu86 _o_	Leu200 _t_	Arg203 _o_	Trp230 _o_
		*	*	*	*	:

Localization of amino acids in predicted IICD topology is indicated with subscripts: i—inner, o—outer, t—transmembrane. Asterisks (*) indicate fully conserved amino acids, colon (:) indicates similar amino acids.

## Supplementary Materials

The following are available online at https://www.mdpi.com/1422-0067/21/21/7860/s1. Table S1. Bacterial strains, plasmids and primers used in this study; Figure S1. Alignment of prepeptide amino acid sequences of BacSJ and its homologs.
