# Supplementary material for: BacSJ—Another Bacteriocin with Distinct Spectrum of Activity that Targets Man-PTS
Source: Int J Mol Sci. 2020 Oct 23;21(21):7860. doi: 10.3390/ijms21217860 (PMC7660280; doi:10.3390/ijms21217860)
Supplement: Supplementary file 1 [file ijms-21-07860-s001.pdf]

Supplementary Materials

Table S1. Bacterial strains, plasmids and primers used in this study

Strains, Plasmids, Primers	Description, NCBI Accession Number	Source (Reference)
	Strains	
Bacillus cereus IBB3390	indicator strain	IBB PAS
Bacillus subtilis BSB1	indicator strain	[1]
Campylobacter jejuni 12	indicator strain	[2]
Campylobacter jejuni 480	indicator strain	[3]
Campylobacter jejuni 81176	indicator strain	[4]
Campylobacter coli 23/1	indicator strain	[2]
Candida albicans CAI-4	indicator strain,	[5]
Carnobacterium maltaromaticum IBB3447	indicator strain	IBB PAS
Enterococcus durans IBB3441	indicator strain	IBB PAS
Enterococcus faecalis IBB3439	indicator strain	IBB PAS
Enterococcus faecalis IBB3444	indicator strain	IBB PAS
Enterococcus faecium LMGT 2783	indicator strain	LMGT NMBU
Enterococcus faecium LMGT 2787	indicator strain	LMGT NMBU
Escherichia coli EC1000	indicator strain	[6]
Escherichia coli TG1	indicator strain	[7]
Lactobacillus johnsonii IBB3155	indicator strain	IBB PAS
Lactobacillus kunkeei AH1	indicator strain	LMGT NMBU
Lactobacillus kunkeei AH38	indicator strain	LMGT NMBU
Lactobacillus kunkeei AH119	indicator strain	LMGT NMBU
Lactobacillus paracasei IBB3418	indicator strain	IBB PAS
Lactobacillus paracasei IBB3425	indicator strain	IBB PAS
Lactobacillus paracasei IBB3426	indicator strain	IBB PAS
Lactobacillus paracasei IBB3427	indicator strain	IBB PAS
Lactobacillus paracasei IBB3428	indicator strain	IBB PAS
Lactobacillus paracasei LOCK 0919	indicator strain, CP005486.1	[8]
Lactobacillus paracasei subsp. *paracasei* IBB3423	indicator strain	[9]
Lactobacillus paraplantarum IBB3438	indicator strain	IBB PAS
Lactobacillus plantarum NC8	indicator strain, AGRI00000000.1	LMGT NMBU [10]
Lactobacillus plantarum WCSF1	indicator strain, AL935263.2	LMGT NMBU [11]
Lactobacillus plantarum IBB3433	indicator strain	IBB PAS
Lactobacillus plantarum IBB3436	indicator strain	IBB PAS
Lactobacillus plantarum subsp. *plantarum* IBB3434	indicator strain	IBB PAS

Lactobacillus rhamnosus GG	indicator strain, AP011548	Dicoflor [12]
Lactobacillus rhamnosus IBB3429	indicator strain	IBB PAS
Lactobacillus rhamnosus LOCK 0900	indicator strain, CP005484.1	LOCK [13]
Lactobacillus rhamnosus LOCK 0908	indicator strain, CP005485.1	LOCK [14]
Lactobacillus salivarius IBB3154	indicator strain	IBB PAS
Lactococcus garvieae IBB3403	indicator strain	IBB PAS
Lactococcus garvieae IBB66	indicator strain	IBB PAS
Lactococcus lactis IBB3404	indicator strain	IBB PAS
Lactococcus lactis IBB3411	indicator strain	IBB PAS
Lactococcus lactis QU5 LMGT 3419	indicator strain	LMBT NMBU
Lactococcus lactis subsp. *cremoris* IBB3409	indicator strain	IBB PAS
Lactococcus lactis subsp. *lactis* IBB3407	indicator strain	IBB PAS
Lactococcus lactis subsp. *lactis* IL1403	indicator strain, host strain, AE005176	INRA [15]
Lactococcus raffinolactis IBB91	indicator strain	IBB PAS
Leuconostoc lactis IBB3446	indicator strain	IBB PAS
Leuconostoc mesenteroides IBB3442	indicator strain	IBB PAS
Leuconostoc mesenteroides IBB3443	indicator strain	IBB PAS
Listeria monocytogenes EGD-e LMGT 2604	indicator strain, AL591824.1	LMGT NMBU [16]
Pediococcus acidilacti LMGT 2002	indicator strain	LMGT NMBU
Pediococcus parvulus IBB3448	indicator strain	IBB PAS
Pediococcus pentosaceus IBB3369	indicator strain	IBB PAS
Pseudomonas aeruginosa ATCC 9027	indicator strain	ATCC
Salmonella typhimurium TT622	indicator strain	[17]
Staphylococcus aureus ATCC 6538	indicator strain	ATCC
Staphylococcus caprae DSM-20608	indicator strain	DSMZ
Staphylococcus delphini DSM-20771	indicator strain	DSMZ
Staphylococcus epidermidis DSM-20044	indicator strain	DSMZ
Staphylococcus hyicus DSM-20459	indicator strain	DSMZ
Staphylococcus intermedius DSM-20373	indicator strain	DSMZ
Staphylococcus lugdunesis DSM-4804	indicator strain	DSMZ
Staphylococcus pseudintermedius DSM-21284	indicator strain	DSMZ
Staphylococcus saprophyticus DSM-18669	indicator strain	DSMZ
Staphylococcus schleiferi DSM-6628	indicator strain	DSMZ
Streptococcus agalactiae IBB123	indicator strain	IBB PAS
Streptococcus agalactiae IBB130	indicator strain	IBB PAS
Streptococcus mitis IBB3449	indicator strain	IBB PAS
Streptococcus sobrinus IBB3450	indicator strain	IBB PAS
Streptococcus parauberis IBB272	indicator strain	IBB PAS
	Lactococcus lactis IL1403 - spontaneous missense mutants**	

LLN1	GarQ^r; Man$^+$; Pro123→His in PtnD	[18]
M3, M8, M9	BacSJ2-8^r; Man$^+$; Gly62→Val in PtnC	This study
M6, M33	BacSJ2-8^r; Man$^+$; Arg200→His in PtnD	This study
M16	BacSJ2-8^r; Man$^+$; Leu83→Phe in PtnD	This study
M19	BacSJ2-8^r; Man$^+$; Phe226→Ser in PtnD	This study
M30	BacSJ2-8^r; Man$^+$; Leu197→Phe in PtnD	This study
	Lactococcus lactis IL1403 derivative strains with deleted *ptnABCD***	
B464	IL1403 strain with *ptnABCD* deletion; Man$^-$; GarABCQ^r	LMBT NMBU [18,19,20]
B488	B464 carrying pNZ9530	LMBT NMBU [18,19,20]
B520	B488 carrying pNZ8037; Man$^-$; GarABCQ^r	LMBT NMBU [18,19,20]
B515	B488 carrying pNZ8037 with *ptnABCD*; Man$^+$; GarAB^r; GarCQ^s	LMBT NMBU [18,19,20]
B538	B488 carrying pNZ8037 with *ptnC*; Man$^-$; GarABCQ^r	LMBT NMBU [18,19,20]
B541	B488 carrying pNZ8037 with *ptnD*; Man$^-$; GarABCQ^r	LMBT NMBU [18,19,20]
B529	B488 carrying pNZ8037 with *ptnCD*; Man$^+$; GarAB^r; GarCQ^s	LMBT NMBU [18,19,20]
B557a	B488 carrying pNZ8037 with *manABCD*; Man$^+$; GarABCQ^s	[19]
B558a	B488 carrying pNZ8037 with *manCD*; Man$^+$; GarABCQ^s	[19]
B561a	B488 carrying pNZ8037 with *manCD* without γ+; Man$^+$; GarABC^r; GarQ^s	[19]
H1	B488 carrying pNZ8037 with *mptCD*; Man$^+$; GarABC^r; GarQ^s	[21]
	Plasmids	
pNZ9530	Em^r, carrying nisin-regulatory *nisRK* genes	[22]
pNZ8037	Cam^r, nisin regulated expression system containing nisin-responsive promoter	[23]
Primers	Nucleotide sequence (5'->3')	
*ptn*Cfor/rev	TCTGACCTCTTTGGTTTG/GCATACGTTCGTAGT	
*ptn*Dfor/rev	AACCTCCAAGCTTCTG/AGCCACAGATTCTCTCC	

*Bacterial strains derived from the Regional Strains and Plasmids Collection of the Institute of Biochemistry and Biophysics, Warsaw, Poland (IBB PAS), from the Pure Cultures Collection of the Institute of Fermentation Technology and Microbiology, Technical University of Lodz, Poland (LOCK), from the dietary supplement Dicoflor, Vitis Pharma, Poland (Dicoflor), the collection of the Laboratory of Microbial Gene Technology, Department of Chemistry, Biotechnology and Food Science, Norwegian University of Life Sciences, Ås, Norway (LMGT NMBU), the Collection of Microorganisms and Cell Cultures, Germany (DSMZ), the collection of the National Institute for Agricultural Research, France (INRA), the American Type Culture Collection (ATCC), and obtained in this study. **Man—mannose phenotype.

Figure S1. Alignment of prepeptide amino acid sequences of BacSJ and its homologs. Fully conserved residues are in red, partially conserved ones in blue. Consensus symbols are: ! - I or V, # is any of DQE. GG motifs are highlighted by grey background. Boxes mark groups of closely related (identical or highly similar) bacteriocins.

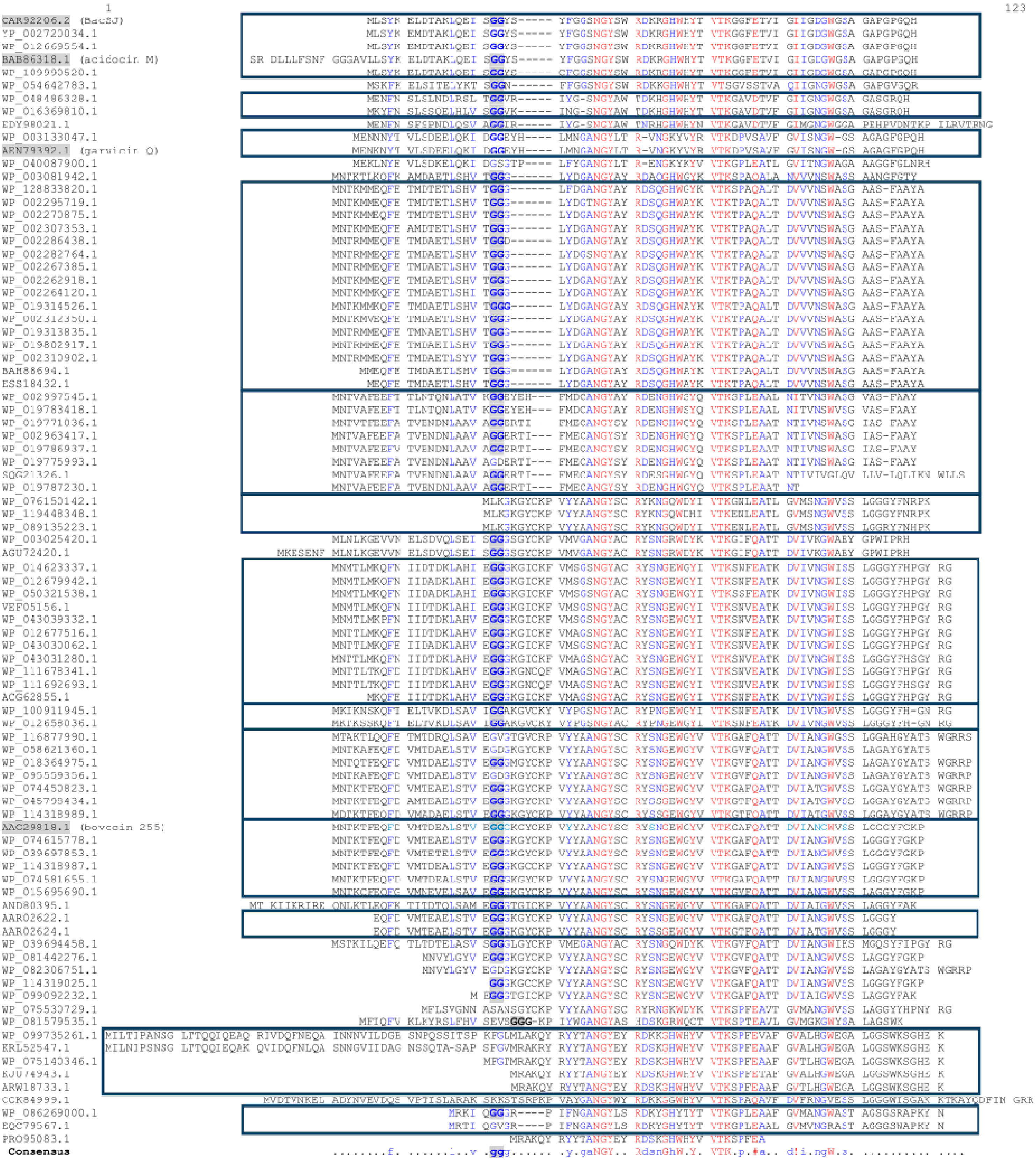

The protein accession numbers and host organisms are: CAR92206.2 - bacteriocin BacSJ (plasmid) [*Lactobacillus*

paracasei subsp. *paracasei* BGSJ2-8]; WP_012669554.1 - bacteriocin [*Lactobacillus paracasei*]; WP_016370066.1 - bacteriocin [*Lactobacillus paracasei*]; BAB86318.1 - acidocin M, partial (plasmid) [*Lactobacillus acidophilus* TK8912]; WP_109990520.1 - bacteriocin [*Lactobacillus paracasei*]; WP_048486328.1 - bacteriocin [*Lactobacillus rhamnosus*]; WP_016369810.1 - hypothetical protein [*Lactobacillus paracasei*]; WP_054642783.1 - hypothetical protein [*Lactobacillus paralimentarius*]; EDY98021.1 - hypothetical protein LRH_09920 [*Lactobacillus rhamnosus* HN001]; WP_003081942.1 - ComC/BlpC family peptide pheromone/bacteriocin [*Streptococcus* macacae]; WP_128833820.1 - ComC/BlpC family peptide pheromone/bacteriocin [*Streptococcus troglodytae*]; WP_002295719.1 - ComC/BlpC family peptide pheromone/bacteriocin [*Streptococcus mutans*]; WP_002270875.1 - ComC/BlpC family peptide pheromone/bacteriocin [*Streptococcus mutans*]; WP_002307353.1 - ComC/BlpC family peptide pheromone/bacteriocin [*Streptococcus mutans*]; WP_002286438.1 - ComC/BlpC family peptide pheromone/bacteriocin [*Streptococcus mutans*]; WP_002282764.1 - ComC/BlpC family peptide pheromone/bacteriocin [*Streptococcus mutans*]; WP_086269000.1 - MULTISPECIES: hypothetical protein [*Enterococcus*]; WP_002264120.1 - ComC/BlpC family peptide pheromone/bacteriocin [*Streptococcus mutans*]; BAH88694.1 - putative bacteriocin peptide precursor [*Streptococcus mutans* NN2025]; WP_019314526.1 - ComC/BlpC family peptide pheromone/bacteriocin [*Streptococcus mutans*]; ESS18432.1 - putative bacteriocin peptide [*Streptococcus mutans* PKUSS-LG01]; WP_002262918.1 - ComC/BlpC family peptide pheromone/bacteriocin [*Streptococcus mutans*]; WP_002267385.1 - ComC/BlpC family peptide pheromone/bacteriocin [*Streptococcus mutans*]; WP_019802917.1 - hypothetical protein [*Streptococcus mutans*]; WP_002310902.1 - ComC/BlpC family peptide pheromone/bacteriocin [*Streptococcus mutans*]; WP_099735261.1 - hypothetical protein [*Lactobacillus plantarum*]; WP_002312350.1 - ComC/BlpC family peptide pheromone/bacteriocin [*Streptococcus mutans*]; WP_002997545.1 - MULTISPECIES: hypothetical protein [*Streptococcus*]; WP_019313835.1 - bacteriocin [*Streptococcus mutans*]; KJU74943.1 - bacteriocin [*Pediococcus damnosus* LMG 28219]; WP_019783418.1 - hypothetical protein [*Streptococcus sobrinus*]; KRL52547.1 - hypothetical protein FD35_GL001890 [*Lactobacillus rossiae* DSM 15814]; ARW18733.1 - hypothetical protein S100892_00127 (plasmid) [*Pediococcus pentosaceus*]; WP_075140346.1 - MULTISPECIES: hypothetical protein [*Pediococcus*]; WP_003133047.1 - bacteriocin [*Lactococcus garvieae*]; AEN79392.1 - prepeptide GarQ (plasmid) [*Lactococcus garvieae*]; EQC79567.1 - hypothetical protein HSIEG1_1895 [*Enterococcus* sp. HSIEG1]; WP_081579535.1 - hypothetical protein [*Leuconostoc carnosum*]; WP_019771036.1 - hypothetical protein [*Streptococcus sobrinus*]; WP_002963417.1 - hypothetical protein [*Streptococcus sobrinus*]; WP_076150142.1 - bacteriocin [*Lactobacillus murinus*]; WP_003025420.1 - MULTISPECIES: hypothetical protein [*Streptococcus*]; AGU72420.1 - hypothetical protein SCRE_0568 [*Streptococcus constellatus* subsp. *pharyngis* C232]; WP_014623337.1 - ComC/BlpC family peptide pheromone/bacteriocin [*Streptococcus equi*]; ACG62855.1 - bacteriocin-like peptide [*Streptococcus equi* subsp. *zooepidemicus* MGCS10565]; WP_012677516.1 - ComC/BlpC family peptide pheromone/bacteriocin [*Streptococcus equi*]; WP_019786937.1 - hypothetical protein [*Streptococcus sobrinus*]; WP_075530729.1 - hypothetical protein [*Lactobacillus ruminis*]; WP_019775993.1 - hypothetical protein [*Streptococcus sobrinus*]; WP_116877990.1 - MULTISPECIES: ComC/BlpC family peptide pheromone/bacteriocin [*Streptococcus*]; AAG29818.1 - bovicin 255 peptide precursor [Streptococcus sp. LRC 0255]; WP_012679942.1 - hypothetical protein [*Streptococcus equi*]; WP_089135223.1 - ComC/BlpC family peptide pheromone/bacteriocin [*Lactobacillus murinus*]; WP_050321538.1 - hypothetical protein [*Streptococcus equi*]; WP_074615778.1 - ComC/BlpC family peptide pheromone/bacteriocin [*Streptococcus equinus*]; AAR02622.1 - bovicin 255 variant, partial [*Streptococcus equinus*]; WP_039694458.1 - ComC/BlpC family peptide pheromone/bacteriocin [*Streptococcus gallolyticus*]; VEF05156.1 - bacteriocin [*Streptococcus equi* subsp. *zooepidemicus*]; PRO95083.1 - ComC/BlpC family peptide pheromone/bacteriocin, partial [*Lactobacillus pentosus*]; WP_015695690.1 - hypothetical protein [*Streptococcus infantarius*]; WP_043031280.1 - ComC/BlpC family peptide pheromone/bacteriocin [*Streptococcus equi*]; WP_058621360.1 - hypothetical protein [*Streptococcus gallolyticus*]; WP_018364975.1 - ComC/BlpC family peptide pheromone/bacteriocin [*Streptococcus caballi*]; WP_043039332.1 - hypothetical protein [*Streptococcus equi*]; WP_040087900.1 - hypothetical protein [*Lactococcus garvieae*]; WP_074450823.1 - ComC/BlpC family peptide pheromone/bacteriocin [*Streptococcus equinus*]; WP_039697853.1 - ComC/BlpC family peptide pheromone/bacteriocin [*Streptococcus equinus*]; WP_095559356.1 - ComC/BlpC family peptide pheromone/bacteriocin [*Streptococcus thermophilus*]; WP_114318987.1 - ComC/BlpC family peptide pheromone/bacteriocin [*Streptococcus gallolyticus*]; WP_074581655.1 - ComC/BlpC family peptide pheromone/bacteriocin [*Streptococcus gallolyticus*]; AND80395.1 - bacteriocin [*Streptococcus pantholopis*]; WP_100911945.1 - bacteriocin [*Streptococcus uberis*]; WP_012658036.1 - MULTISPECIES: bacteriocin [*Streptococcus*]; WP_114319025.1 - ComC/BlpC family peptide pheromone/bacteriocin, partial [*Streptococcus*

gallolyticus]; WP_045798434.1 - ComC/BlpC family peptide pheromone/bacteriocin [*Streptococcus equinus*]; WP_119448348.1 - ComC/BlpC family peptide pheromone/bacteriocin [*Lactobacillus murinus*]; AAR02624.1 - bovicin 255 variant, partial [*Streptococcus equinus*]; WP_111678341.1 - ComC/BlpC family peptide pheromone/bacteriocin [*Streptococcus equi*]; WP_081442276.1 - bacteriocin [*Streptococcus infantarius*]; WP_111692693.1 - ComC/BlpC family peptide pheromone/bacteriocin [*Streptococcus equi*]; WP_043030062.1 - ComC/BlpC family peptide pheromone/bacteriocin [*Streptococcus equi*]; WP_099092232.1 - ComC/BlpC family peptide pheromone/bacteriocin [*Streptococcus pantholopis*]; WP_114318989.1 - ComC/BlpC family peptide pheromone/bacteriocin [*Streptococcus gallolyticus*]; WP_082306751.1 - bacteriocin [*Streptococcus gallolyticus*]; SQG21326.1 - bacteriocin peptide precursor [*Streptococcus sobrinus*]; WP_019787230.1 - hypothetical protein, partial [*Streptococcus sobrinus*]; CCK84999.1 - Bovicin 255 peptide [*Lactobacillus equicursoris* DSM 19284 = JCM 14600 = CIP 110162].

References

1. Nicolas, P.; Mäder, U.; Dervyn, E.; Rochat, T.; LeDuc, A.; Pigeonneau, N.; Bidnenko, E.; Marchadier, E.; Hoebeke, M.; Aymerich, S.; et al. Condition-dependent transcriptome reveals high-level regulatory architecture in *Bacillus subtilis*. *Science* **2012**, *335*, 1103–1106, doi:10.1126/science.1206848.
2. Wyszyńska, A.; Raczko, A.; Lis, M.; Jagusztyn-Krynicka, E.K. Oral immunization of chickens with avirulent *Salmonella* vaccine strain carrying *C. jejuni* 72Dz/92 *cjaA* gene elicits specific humoral immune response associated with protection against challenge with wild-type *Campylobacter*. *Vaccine* **2004**, *22*, 1379–1389, doi:10.1016/j.vaccine.2003.11.001.
3. Grabowska, A.D.; Wandel, M.P.; Łasica, A.M.; Nesteruk, M.; Roszczenko-Jasinska, P.; Wyszyńska, A.K.; Godlewska, R.; Jagusztyn-Krynicka, E.K. *Campylobacter jejuni dsb* gene expression is regulated by iron in a Fur-dependent manner and by a translational coupling mechanism. *BMC Microbiol.* **2011**, *11*, 166, doi:10.1186/1471-2180-11-166.
4. Korlath, J.A.; Osterholm, M.T.; Judy, L.A.; Forfang, J.C.; Robinson, R.A. A point-source outbreak of campylobacteriosis associated with consumption of raw milk. *J. Infect. Dis.* **1985**, *152*, 592–596, doi:10.1093/infdis/152.3.592.
5. Fonzi, W.A.; Irwin, M.Y. Isogenic strain construction and gene mapping in *Candida albicans*. *Genetics* **1993**, *134*, 717–728.
6. Leenhouts, K.; Buist, G.; Bolhuis, A.; Berge, A.T.; Kiel, J.; Mierau, I.; Dabrowska, M.; Venema, G.; Kok, J. A general system for generating unlabelled gene replacements in bacterial chromosomes. *Mol. Genet. Genom.* **1996**, *253*, 217–224, doi:10.1007/s004380050315.
7. Gibson, T.J. *Studies on the Epstein-Barr Virus Genome*; Cambridge University: Cambridge, UK, 1984.
8. Koryszewska-Bagińska, A.; Aleksandrzak-Piekarczyk, T.; Bardowski, J. Complete genome sequence of the probiotic strain *Lactobacillus casei* (formerly *Lactobacillus paracasei*) LOCK919. *Genome Announc.* **2013**, *1*, 1, doi:10.1128/genomea.00758-13.
9. Koryszewska-Bagińska, A.; Gawor, J.; Nowak, A.; Grynberg, M.; Aleksandrzak-Piekarczyk, T. Comparative genomics and functional analysis of a highly adhesive dairy *Lactobacillus paracasei* subsp. *paracasei* IBB3423 strain. *Appl. Microbiol. Biotechnol.* **2019**, *103*, 7617–7634, doi:10.1007/s00253-019-10010-1.
10. Axelsson, L.; Rud, I.; Naterstad, K.; Blom, H.; Renckens, B.; Boekhorst, J.; Kleerebezem, M.; Van Hijum, S.; Siezen, R.J. Genome sequence of the naturally plasmid-free *Lactobacillus plantarum* strain NC8 (CCUG 61730). *J. Bacteriol.* **2012**, *194*, 2391–2392, doi:10.1128/jb.00141-12.
11. Kleerebezem, M.; Boekhorst, J.; Van Kranenburg, R.; Molenaar, D.; Kuipers, O.P.; Leer, R.; Tarchini, R.; Peters, S.A.; Sandbrink, H.M.; Fiers, M.W.E.J.; et al. Complete genome sequence of *Lactobacillus plantarum* WCFS1. *Proc. Natl. Acad. Sci. USA* **2003**, *100*, 1990–1995, doi:10.1073/pnas.0337704100.
12. Morita, H.; Toh, H.; Oshima, K.; Murakami, M.; Taylor, T.D.; Igimi, S.; Hattori, M. Complete genome sequence of the probiotic *Lactobacillus rhamnosus* ATCC 53103. *J. Bacteriol.* **2009**, *191*, 7630–7631, doi:10.1128/jb.01287-09.
13. Aleksandrzak-Piekarczyk, T.; Koryszewska-Bagińska, A.; Bardowski, J. Genome sequence of the probiotic strain *Lactobacillus rhamnosus* (formerly *Lactobacillus casei*) LOCK900. *Genome Announc.* **2013**, *1*, doi:10.1128/genomea.00640-13.
14. Koryszewska-Bagińska, A.; Bardowski, J.; Aleksandrzak-Piekarczyk, T. Genome sequence of the probiotic strain *Lactobacillus rhamnosus* (formerly *Lactobacillus casei*) LOCK908. *Genome Announc.* **2014**, *2*, doi:10.1128/genomea.00120-14.

15. Bolotin, A.; Wincker, P.; Mauger, S.; Jaillon, O.; Malarme, K.; Weissenbach, J.; Ehrlich, S.D.; Sorokin, A. The complete genome sequence of the lactic acid bacterium *Lactococcus lactis* ssp. *lactis* IL1403. *Genome Res.* **2001**, *11*, 731–753, doi:10.1101/gr.gr-1697r.
16. Glaser, P.; Frangeul, L.; Buchrieser, C.; Rusniok, C.; Amend, A.; Baquero, F.; Berche, P.; Bloecker, H.; Brandt, P.; Chakraborty, T.; et al. Comparative genomics of *Listeria* species. *Science* **2001**, *294*, 849–852, doi:10.1126/science.1063447.
17. Chumley, F.G.; Menzel, R.; Roth, J.R. Hfr formation directed by Tn10. *Genetics* **1979**, *91*, 639–655.
18. Tymoszewska, A.; Diep, D.B.; Wirtek, P.; Aleksandrzak-Piekarczyk, T. The non-lantibiotic bacteriocin garvicin Q targets Man-PTS in a broad spectrum of sensitive bacterial genera. *Sci. Rep.* **2017**, *7*, 8359, doi:10.1038/s41598-017-09102-7.
19. Tymoszewska, A.; Diep, D.B.; Aleksandrzak-Piekarczyk, T. The extracellular loop of Man-PTS subunit IID is responsible for the sensitivity of *Lactococcus garvieae* to garvicins A, B and C. *Sci. Rep.* **2018**, *8*, 15790, doi:10.1038/s41598-018-34087-2.
20. Diep, D.B.; Skaugen, M.; Salehian, Z.; Holo, H.; Nes, I.F. Common mechanisms of target cell recognition and immunity for class II bacteriocins. *Proc. Natl. Acad. Sci. USA* **2007**, *104*, 2384–2389, doi:10.1073/pnas.0608775104.
21. Kjos, M.; Salehian, Z.; Nes, I.F.; Diep, D.B. An extracellular loop of the mannose phosphotransferase system component IIC is responsible for specific targeting by class IIa bacteriocins. *J. Bacteriol.* **2010**, *192*, 5906–5913, doi:10.1128/jb.00777-10.
22. Kleerebezem, M.; Beerthuyzen, M.M.; Vaughan, E.E.; De Vos, W.M.; Kuipers, O.P. Controlled gene expression systems for lactic acid bacteria: transferable nisin-inducible expression cassettes for *Lactococcus*, *Leuconostoc*, and *Lactobacillus* spp. *Appl. Environ. Microbiol.* **1997**, *63*, 4581–4584, doi:10.1128/aem.63.11.4581-4584.1997.
23. De Ruyter, P.G.; Kuipers, O.P.; De Vos, W.M. Controlled gene expression systems for *Lactococcus lactis* with the food-grade inducer nisin. *Appl. Environ. Microbiol.* **1996**, *62*, 3662–3667, doi:10.1128/aem.62.10.3662-3667.1996.
